# Diagnostic value of structural, functional and effective connectivity in bipolar disorder

**DOI:** 10.1111/acps.13742

**Published:** 2024-08-13

**Authors:** Teodora M. Gencheva, Bozhidar V. Valkov, Sevdalina S. Kandilarova, Michael H. J. Maes, Drozdstoy S. Stoyanov

**Affiliations:** ^1^ Faculty of Medicine Medical University of Plovdiv Plovdiv Bulgaria; ^2^ Department of Psychiatry and Medical Psychology, and Research Institute Medical University of Plovdiv Plovdiv Bulgaria; ^3^ Department of Psychiatry, Faculty of Medicine Chulalongkorn University Bangkok Thailand; ^4^ Sichuan Provincial Center for Mental Health, Sichuan Provincial People's Hospital, School of Medicine University of Electronic Science and Technology of China Chengdu China; ^5^ Research and Innovation Program for the Development of MU – PLOVDIV – (SRIPD‐MUP), Creation of a Network of Research Higher Schools, National Plan For Recovery and Sustainability, European Union – NextGenerationEU Plovdiv Bulgaria

**Keywords:** bipolar disorder, effective connectivity, fMRI, functional connectivity, structural connectivity

## Abstract

**Introduction:**

The aim of this systematic review is to assess the functional magnetic resonance imaging (fMRI) studies of bipolar disorder (BD) patients that characterize differences in terms of structural, functional, and effective connectivity between the patients with BD, patients with other psychiatric disorders and healthy controls as possible biomarkers for diagnosing the disorder using neuroimaging.

**Methods:**

Following the Preferred Reporting Items for Systematic Reviews and Meta‐Analyses (PRISMA), guidelines a systematic search for recent (since 2015) original studies on connectivity in bipolar disorder was conducted in PUBMED and SCOPUS.

**Results:**

A total of 60 studies were included in this systematic review: 20 of the structural connectivity, 33 of the functional connectivity, and only 7 of the studies focused on effective connectivity complied with the inclusion and exclusion criteria.

**Discussion:**

Despite the great heterogeneity in the findings, there are several trends that emerge. In structural connectivity studies, the main abnormalities in bipolar disorder patients were in the frontal gyrus, anterior, as well as posterior cingulate cortex and differences in emotion and reward‐related networks. Cerebellum (vermis) to cerebrum functional connectivity was found to be the most common finding in BD. Moreover, prefrontal cortex and amygdala connectivity as part of the rich‐club hubs were often reported to be disrupted. The most common findings based on effective connectivity were alterations in salience network, default mode network and executive control network.

Although more studies with larger sample sizes are needed to ascertain altered brain connectivity as diagnostic biomarker, there is a perspective that the method could be used as a single marker of diagnosis in the future, and the process of adoption could be accelerated by using approaches such as semiunsupervised machine learning.

**Summations**

- As bipolar disorder (BD) is often misdiagnosed in 60% of the cases, it is necessary to identify biomarkers that allow accurate and objective diagnosis.
- Functional magnetic resonance imaging (fMRI) demonstrated abnormal connectivity in three major neural networks: salience network (SN), executive control network (ECN), and default mode network (DMN) in patients with bipolar disorder compared with healthy controls.
- Semiunsupervised machine learning could be used in fMRI studies in the future to find patient subgroup clusters to address the issue with small sample sizes in patient groups, such as BD.

**Limitations**

- Due to sample size limitations and the clinical heterogeneity of bipolar disorder, no definite conclusions can be made about biomarkers that could be used in the future for diagnosing BD.
- There are low levels of evidence of any potential biomarker as the number of convergent studies is small and the results are often not replicated.
- A serious limitation is the heterogeneity of the methods employed and the lack of an atlas of the normal brain structure that functional connectivity is mapped on. There is no universally adapted methodology for data processing, which is the reason why we observe a large heterogeneity which undermines the validity and translation of the data. Amplitude of low‐frequency fluctuation (ALFF) was included as a method, although according to some authors it is a measure that does not specifically represent functional connectivity rather a spontaneous neural activity of specific regions.
- Bias has not been assessed in the systematic review, as well bias has not been assessed in other recently published studies.^1^, ^2^

## 1 INTRODUCTION

Bipolar disorder (BD), previously known as manic depressive illness, is a severe and chronic mood disorder characterized by alternating or concurrent episodes of depression, mania, and hypomania.^3^ Clinical criteria are still used for the diagnosis of mental disorders, and no biomarker has yet been approved for use.^4^ Notably, 60% of patients with BD are initially diagnosed with unipolar depression.^5^ Although 20% of patients receive a correct diagnosis of BD during a depressive episode within the first year of seeking therapy, this highlights the critical need for new diagnostic methods^6^. Differentiating unipolar depression from bipolar depression is essential to prevent misdiagnosis and inappropriate treatment, which can result in adverse outcomes, such as triggering manic episodes in BD patients.

Functional magnetic resonance imaging (fMRI), which is dependent on blood oxygen levels, reveals alterations in deoxyhemoglobin concentration due to task‐induced or spontaneous brain metabolic regulation.^7^, ^8^ In addition, multimodal brain research can help to clarify the biological significance of various imaging measurements and the intricate interactions between morphological, functional, and physiological changes in the brain.^9^

The term brain connectivity encompasses various approaches, including, anatomical connections (structural connectivity), statistical dependencies (functional connectivity [FC]), and causal interactions (effective connectivity). FC refers to the temporal coincidence of spatially distant neurophysiological processes. In other words, if there is a statistical correlation between the activity measures that are reported for two regions, these regions are said to exhibit FC.^10^ Effective connectivity describes the influences (inhibitory or excitatory) of one region over another, either at rest or during a given task.^11^

Current research on the use of fMRI to identify biomarkers for BD patients suggests that this technology has the potential to play a clinically important role in both the diagnostic and treatment procedures.^12^ Recently, fMRI has been employed alongside advanced analytical methods, such as machine learning, raising hopes for discovering connectivity‐based biomarkers as digital tools in future mental healthcare.

Previous findings suggest disrupted integrity of commissural fibers and white matter in the anterior paralimbic structures in individuals with BD.^13^ Additionally, researchers reported structural and FC anomalies between the prefrontal cortices and subcortical regions in the emotion regulation circuitry among participants with bipolar I disorder.^14^ The involvement of a disconnected corticolimbic network in both the onset and persistence of BD has been emphasized; highlighting its potential as a diagnostic biomarker for the condition.^15^ A review published in 2019 summarizes reports indicating that alterations in structural, functional, and effective connectivity in BD selectively affect circuits supporting executive, cognitive, and emotional processes. The authors conclude that dynamic instabilities in interoceptive circuits, which subsequently alter fear circuitry and cognitive control systems, are the root cause of the affective dysregulation associated with BD.^16^

## 2 AIM

The aim of the current systematic review was to summarize the evidence of potential MRI based biomarkers for diagnosing BD over the past 10 years, focusing on structural, functional, and effective connectivity. Additionally, this review aims to summarize findings regarding differences between BD patients, patients with major depressive disorder (MDD), and other psychiatric disorders. Previous systematic reviews have focused on narrower topics, such as neural circuitry of facial emotion processing,^17^, ^18^ or on multiple diagnoses.^2^, ^19^ We have identified a shortage of systematic reviews addressing structural, functional, and effective connectivity collectively.^2^, ^20^

Therefore, we believe there is a need for a comprehensive article discussing the potential future diagnosis of BD using MRI. We found only one systematic review dedicated to BD, which was notably published in 2013.^2^ The most consistent conclusion of that review is that there are no differences between individuals with BD and controls in terms of the default mode network (DMN), frontoparietal network (FPN), and salience network (SN) regarding their resting‐state FC. The main limitation of that review was the heterogeneity of the analysis methods used. The specific focus of the current review is the potential for objective diagnostic classification of BD based on MRI‐derived brain connectivity measures.

## 3 MATERIALS AND METHODS

Our aim was to evaluate literature on structural, functional, and effective connectivity in BD published between January 2015 and January 2024. Two authors independently searched PubMed and SCOPUS databases for relevant articles using combinations of keywords in the following search string: (structural connectivity[Title/Abstract]) AND (bipolar disorder[Title/Abstract]); (functional connectivity[Title/Abstract]) AND (bipolar disorder[Title/Abstract]); (effective connectivity[Title/Abstract]) AND (bipolar disorder[Title/Abstract]). Тhe two authors independently screened the retrieved titles and abstract for eligibility. Additionally, the reference lists of the selected articles and relevant review articles were manually examined to include additional studies.

Inclusion criteria were as follows: human research published in English in 2015 or later, peer‐reviewed original articles on structural, functional, and effective connectivity in BD (both in remission and during episodes) compared with healthy controls or to patients with schizophrenia or MDD (in accordance with DSM/ICD diagnostic criteria) as psychiatric comparison groups. For FC studies, network analysis, seed‐based, and ROI studies were all eligible. Exclusion criteria were review articles; studies based on comparison between patient groups without healthy controls. The studies were assessed by two blinded raters who determined if they complied with the inclusion and exclusion criteria. The systematic review was conducted in accordance with the Preferred Reporting Items for Systematic Reviews and Meta‐Analyses (PRISMA) guidelines.

## 4 RESULTS

Using the keywords “structural connectivity” and “bipolar disorder” in the search string (structural connectivity[Title/Abstract]) AND (bipolar disorder [Title/Abstract]), 44 results were found. Hits were first screened at the title/abstract level, followed by an assessment of the abstracts and full texts of the remaining articles. Finally, 20 of the screened articles were included in the review. For the keywords “functional connectivity,” “fMRI,” and “bipolar disorder” in the search string ((functional connectivity[Title/Abstract]) AND (fmri[Title/Abstract])) AND (bipolar disorder[Title/Abstract]), 163 results were found. Of those, only 33 complied with the inclusion/exclusion criteria.

**FIGURE 1:**
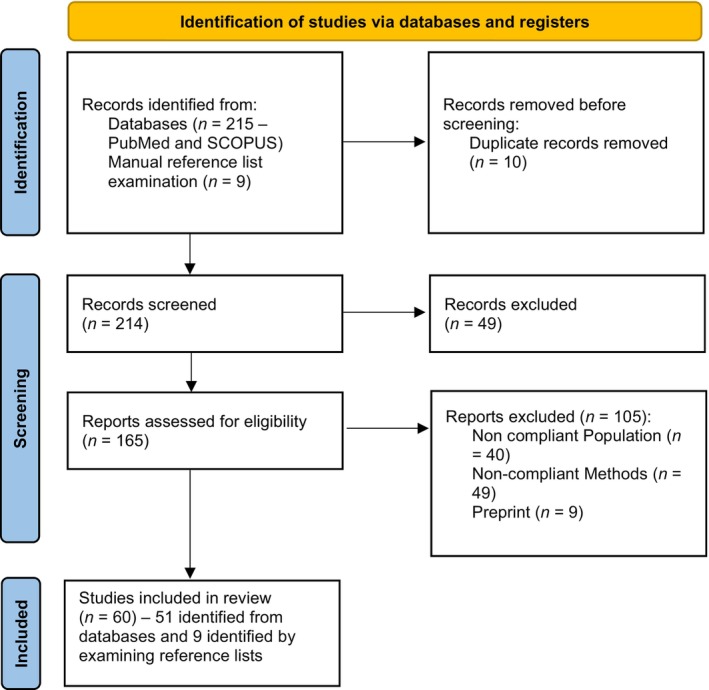
Preferred Reporting Items for Systematic Reviews and Meta‐Analyses (PRISMA) flow chart.^21^

Searching with the keywords: “effective connectivity” and “bipolar disorder” in the following search string: (effective connectivity[Title/Abstract]) AND (bipolar disorder[Title/Abstract]) yielded 17 results, and after the assessment of the abstracts only 7 articles were included in the review. We also manually examined the reference lists of the selected articles and relevant review articles to include additional relevant studies. Nine out of all 60 articles included were identified using this method. The details of the search strategy are presented in Figure 1 (PRISMA flow chart).

### 4.1 Structural connectivity

There were 20 articles identified that complied with our inclusion and exclusion criteria. A detailed description of the studies is provided in Table 1. The majority of the studies used diffusion tensor imaging (DTI) and diffusion‐weighted imaging (DWI) methods, in samples of 80 to 1700 subjects. Most of the studies reported divergent findings. Compared with healthy individuals, BD patients exhibited a lower clustering coefficient in the left occipital and right parietal regions. The clustering coefficient of left anterior cingulate cortex (ACC) was modestly negatively correlated with the score on Young mania rating scale.^36^

**TABLE 1:** Structural connectivity.

Author, year	Aim	Method	Population	Main results
BD versus HC				
Forde et al. (2015)^22^	Investigation of connectivity abnormalities in multiply affected BD type I families with structural brain network analysis; assessment of the utility of dysconnectivity as a biomarker and its endophenotypic potential	DWI	19 patients with BD; 21 of their first; degree unaffected relatives; 18 unrelated healthy controls	Group effect in the middle frontal gyrus on the left and the right medial superior frontal gyrus in BD; impaired anatomical connection in discrete frontal regions in BD
Martino et al. (2016)^23^	Relationship between structural connectivity (SC) and functional connectivity (FC) in the cingulum BD and its various phases	DTI	61 patients with BD; 42 healthy controls	↓ FC between the anterior and posterior parts of the cingulum in manic BD patients compared with depressed BD patients and HC; ↓ SC of the cingulum bundle in manic patients compared with HC
Jeganathan et al. (2018)^24^	Disturbances in the neurocircuitry underlying cognitive‐emotional control in BD and in unaffected relatives at high genetic risk (HR)	DWI	84 high genetic risk (HR) individuals; 38 patients with BD; 96 healthy controls	Impairments in network controllability (left parahippocampal, left middle occipital, left superior frontal, right inferior frontal, and right precentral gyri) in BD. Deficits in BD in a subnetwork (left superior and inferior frontal gyri, postcentral gyrus, and insula)
Spuhler et al. (2018)^25^	Investigation of the utility of a novel diffusion MRI metric, entropy of diffusion, in the search for BD biomarkers	DTI	9 medication‐free patients with BD; 9 healthy controls	Significantly lower mean diffusivity entropy in temporal pole in BD
Nabulsi et al. (2019)^26^	A permutation‐based statistical technique in conjunction with whole‐brain and rich‐club connectivity, examination of topological variance in euthymic BD patients compared with healthy individuals	DWI	40 patients with BD; 45 healthy controls	BD dysconnectivity impacting the communication inside and between emotion‐regulatory and reward‐related subsystems (subject‐specific cortico‐subcortical nontensor‐based connectome map)
Yang et al. (2019)^27^	Identification of the brain connectivity alterations in BD by capturing the latent nexus in multimodal neuroimaging data	DWI	83 patients with BD 94 healthy controls	Anterior cingulate cortex (ACC) and superior medial prefrontal cortex (sMPFC) contribute the most to BD diagnosis. ↓ FC ACC‐sMPFC in BD
Nabulsi et al. (2020)^28^	Independent functional subsystems global integration and their relation with anatomical cortico‐subcortical networks as a key to understanding how the human brain's architecture constrains functional interactions and underpins abnormalities of mood and emotion, particularly in BD	DTI	41 patients with BD; 56 healthy controls	Fronto‐limbic, default‐mode, and fronto‐parietal subsystems connections in BD (not detectable on a global scale)
Jiang et al. (2020)^29^	Structure connectivity constraining functional connectivity; structural–functional coupling as a more sensitive biomarker to detect subtle brain abnormalities than any single modality in BD patients with a current major depressive episode and a suicide attempt	DWI	191 patients with BD; 113 healthy controls	↓ SC—central‐temporal structural connection in BD; ↑ FC frontal‐temporal in BD; ↓ structural‐functional coupling
Nabulsi et al. (2022)^30^	Degree of neuroanatomical dysconnectivity in a sizable representative group of BD patients using multisite graph theory analysis	DWI	109 patients with BD; 103 healthy controls	Longer typical path length in BD; ↓ connected left frontotemporal network in BD; ↑ rich‐club dysconnectivity in BD; emotion and reward networks dysconnectivity in BD
BD versus SCZ				
Dong et al. (2017)^31^	Identification of the shared or disorder‐specific structural abnormalities between SCZ and BD	DTI	BD: 23 datasets with 705 patients with BD versus 679 healthy controls; SCZ: 24 datasets with 754 patients versus 775 healthy controls	↓ Fractional anisotropy (FA) in the genu of the corpus callosum (CC) and posterior cingulum fibers in BD and SCZ
Ji et al. (2019)^32^	Superficial white matter microstructure in vivo in both SCZ and BD	DWI	32 patients with BD; 31 patients with SCZ; 54 healthy controls	Generalized fractional anisotropy (gFA) deficits in BD and SCZ compared with HCs in pars opercularis, insula, anterior cingulate, and precentral gyrus
BD versus MDD				
Cyprien et al. (2016)^33^	Investigation of the specific impact of suicidal behavior on corpus callosum (CC) integrity in mood disorders	DTI	41 patients with BD; 50 patients with MDD; 30 healthy controls	FA values for all CC regions—↓ in BD than in HC
Cha et al. (2022)^34^	Classification of BD and MDD using graph properties of diffusion‐weighted imaging (DWI)‐based structural connectome	DWI	60 patients with BD; 95 patients with MDD; 74 healthy controls	↑ TS‐based connectivity in the left SFG in BD; ↑ FA‐based connectivity in the right middle anterior cingulate area in BD compared with the MDD‐group
Between groups comparisons				
Repple et al. (2022)^35^	Determine unique anomalies in connectivity to each of the following conditions: SCZ, BD, or MDD and which are common to affective and psychotic disorders	DWI	720 patients with MDD; 112 patients with BD; 69 patients with SCZ; 842 healthy controls	Substantial differences between MDD, BD, SCZ, HCs, a converging pattern of changes between diagnoses. Machine learning algorithms were able to distinguish between individual diagnoses from HC, but not between different illnesses

Abbreviations: BD, bipolar disorder; DTI, diffusion tensor imaging; HC, healthy control; MDD, major depressive disorder.

Schizophrenia (SCZ), BD, and MDD demonstrate both distinct and shared signs of structural white matter dysconnectivity, resulting in decreased network efficiency in each group. Subnetwork analysis identified variances across the three illnesses and a core of afflicted edges shared by all three. Machine learning algorithms were able to distinguish between each diagnosis from healthy controls (HC), but not between different conditions.^35^

A DWI study conducted to examine the superficial white matter microstructure in patients with both BD and SCZ revealed generalized fractional anisotropy reductions in bundles connecting pars opercularis, insula, anterior cingulate, and precentral gyrus—regions involved in language processing, mood regulation, working memory, and motor function. This finding was attributed to the short U‐fibers, which are susceptible to the pathological processes associated with severe psychiatric diseases.^32^ A bimodal connectivity study revealed lower left parietal structural connection in BD and SCZ patients compared with HCs.^37^

Schizophrenia and BD showed the opposite correlations between network features and cognition, particularly concerning working memory, attention, and language. Compared with BD, the SCZ group's network of structural and functional connections appeared more fragmented and inefficient. Both locally and globally, the network was different but tightly related with cognitive function, suggesting that the mechanisms underlying cognitive deficits in SCZ and BD may be distinct from one another.^38^

According to a recent study, the structural connectome is the primary factor influencing brain dysfunction, and structural‐functional coupling may serve as a useful trait‐like biomarker for predicting suicide in BD patients. Compared with non‐suicidal patients, individuals with BD who had attempted suicide exhibited considerably lower central‐temporal structural connectivity, higher frontal‐temporal FC, and lower structural‐functional coupling.^29^

Convergent findings highlight a few trends. Abnormalities in BD patients were found in emotion‐regulatory and reward‐related subsystems. Fronto‐temporolimbic nodes and basal ganglia were the primary drivers of greater centrality in females compared with males. A neuroanatomical model of BD dysconnectivity, which differentially impacts communication within and between emotion‐regulatory and reward‐related subsystems, is presented by the subject‐specific cortico‐subcortical non‐tensor‐based connectome map.^26^

In a multicenter study using graph theory analysis, the BD group demonstrated higher rich‐club dysconnectivity, a weakly connected left frontotemporal network, and a longer characteristic path length than the HCs. This multisite research further links BD to dysconnectivity in reward and emotion networks.^30^

Another DWI study attempted to evaluate the machine learning categorization of MDD and BD and found that the communicability efficiency of the left superior frontal gyrus (SFG) was considerably higher in BD than in MDD. Specifically, the BD group showed better communicability efficiency in the left SFG and fractional anisotropy (FA)‐based connectivity in the right middle anterior cingulate area compared with the MDD group. The graph characteristics of DWI‐based connectivity could distinguish between BD and MDD participants with an overall accuracy of 68%.^34^

FMRI analysis of a cohort suggested that certain primarily frontal regions might be endophenotypic. Analysis of these regions indicated a significant group effect in the left middle frontal gyrus and right medial superior frontal gyrus, driven by lower organization in BD patients compared with HC.^22^ The superior medial prefrontal cortex (sMPFC) and ACC showed strongest correlation with the diagnosis of BD. The ACC‐sMPFC functional link was consistently found to be considerably reduced in BD patients as compared to HCs.^27^

Compared with controls, patients with BD showed enhanced connectivity within a frontotemporal subnetwork and decreased connectivity across a subnetwork containing frontolimbic and posterior‐occipital functional connections.^28^ Although occasionally linked to mood state or psychosis, altered interactions between the amygdala and medial prefrontal cortex (MPFC) regions have been associated with BD, though these interactions are less consistently related to core symptoms. Additionally, there have been reports of altered interactions between the medial and lateral ventral PFC in BD, which may impact estimates of the connection between the amygdala and vlPFC.

Studies on FC in BD have revealed altered connections between the amygdala and PFC.^39^ In a recent study, a subnetwork comprising the insula, postcentral gyrus, and left superior and inferior frontal gyri showed widespread abnormalities in people with BD. A right‐lateralized subnetwork containing connections between the superior temporal pole, putamen, caudate nucleus, dorsomedial and ventrolateral prefrontal cortex, exhibited deficiencies in controllability in HR individuals.^24^

Investigating differences between structural (SC) and FC in manic and depressed BD patients, as well as controls, yielded several findings. Firstly, manic individuals showed decreased FC between the anterior and posterior regions of the cingulum compared with depressed patients and HC. Secondly, compared with HC, there was a lower SC of the cingulum bundle in manic individuals, particulary in its anterior region. Lastly, changes in the cingulum structural connectivity (but not FC) were associated with neurocognitive deficiencies in sustained attention in BD, whereas changes in the cingulum FC (but not SC) were correlated with clinical severity scores.^23^

One potential vulnerability indicator for BD is decreased fractional anisotropy (FA). A meta‐analysis revealed that, the genu of the corpus callosum (CC) and the posterior cingulum fibers are two locations with substantial reductions in FA, which indicate aberrant water diffusion in BD and SCZ. The two illnesses did not significantly differ from one another. The findings revealed similar deficits of FA at the genu of the CC and left posterior cingulum fibers, indicating that a shared brain circuit malfunction may be responsible for the phenotypic overlap observed.^31^, ^33^, ^40^ Another potential indicator is mean diffusivity (MD) entropy, which can distinguish bipolar patients from controls with high accuracy. MD was found to be considerably lower in the temporal pole in BD.^25^

### 4.2 Functional connectivity

There were 33 articles identified that complied with our inclusion and exclusion criteria. A detailed description of the studies is provided in Table 2. The majority of the studies used resting‐state FC as a method, and the sample varies between 20 and 200 BD patients and HC, as well as MDD and SCZ patients. Moreover, the BD group demonstrated weaker associations between cognitive empathy and the resting‐state functional connectivity (rsFC) of the temporal–parietal junction with the parahippocampus, cerebellum, and fusiform gyrus compared with HC. These results shed light on the underlying brain mechanisms of empathy deficits in BD patients.^49^

**TABLE 2:** Functional connectivity.

Author, year	Aim	Method	Population	Main results
BD versus HC				
Altinay et al. (2016)^41^	Identification of striatal functional activation and connectivity abnormalities in BDI and BDI	FC	60 patients with BD; 30 healthy controls	BDI—connectivity abnormalities of associative and limbic caudate; BDI—connectivity abnormalities of associative and somatosensory subregions of the putamen
Tseng et al. (2016)^42^	Examining unconscious face emotion processing in patients with BD	FC	14 patients with BD; 14 healthy controls	↓ FC between amygdala and ventromedial prefrontal cortex (vmPFC) in BD
Zhao et al. (2017)^43^	Assessing whether BD patients show abnormal effective connectivity from the prefrontal areas to the amygdala during effortful emotion regulation	FC, DCM	23 patients with BD; 17 healthy controls	↓ Modulatory effect on the connection from the DLPFC to the amygdala in BD
Li G et al. (2018)^44^	To determine changes of the amygdala in patients with euthymic BD	rsFC, ALFF	21 patients with BD; 28 healthy controls	Altered amygdala FC and its connections to other brain areas in BD
Sobczak et al. (2020)^45^	Investigation of the baseline brain activity in euthymic bipolar disorder patients in regard to suicide risk	rsFC	20 patients with BD; 21 healthy controls	↓ FC between regions involved in the salience network in BD
Li et al. (2021)^46^	Investigation of changes in rsFC between the vermis and cerebral regions	rsFC	30 patients with BD; 28 healthy controls	↓ rsFC between the anterior vermis and the middle cingulate cortex and between the whole vermis and the ventral prefrontal cortex (VPFC) in BD
Zhang et al. (2021)^47^	To examine, whether the abnormality in cortical‐striatal neural circuits appears in patients with BD while in euthymic state	rsFC, ALFF	65 patients with BD; 85 healthy controls	↑ rsFC and ALFF in the right caudate/putamen along with ↑ rsFC in the right inferior parietal lobe in BD
Olivito et al. (2022)^48^	To investigate FC between the cerebellum (dentate nucleus) and the cerebrum	rsFC	30 patients with BD; 37 healthy controls	Impaired dentate–cerebral connection affect anterior limbic network areas especially linked to BD's hypomanic states
Liang et al. (2022)^49^	To examine the empathy‐related rsFC in BD patients	rsFC	37 patients with BD; 42 healthy controls	↓ rsFC of the temporal–parietal junction (TPJ) with the fusiform gyrus, the cerebellum, and the parahippocampus in BD
Zhu W et al. (2022)^50^	Investigation of possible correlations between executive dysfunction and the abnormal FC of the sensory motor network (SMN) in BDI patients	FC	20 patients with BD; 20 healthy controls	↑ FC between the SMN and the default mode network (DMN) and the dorsal attention network (DAN) in BD; ↓ FC between the DAN and the auditory network (AN) and between the SMN and DMN in BD
Ping et al. (2022)^51^	Investigation of the voxel‐wise change of functional connectivity patterns in BD patients using publicly available data from the UCLACNP LA5c study	rsFC	45 patients with BD; 115 healthy controls	Aberrant whole‐brain FC homogeneity and functional connections in the brain regions involved the DMN and SMN networks in BD
Ghaznavi et al. (2023)^52^	Aimeed to study the connections in default mode network (DMN) in patients with BD	FC	15 patients with BD; 17 healthy controls	Different patterns of FC within the DMN in BD act as subservient for both positive and negative rumination. Furthermore, after positive rumination, the PCC and MPFC are important brain areas that process positive self‐relevant qualities
Saleem et al. (2023)^53^	Assessment of functional connectivity of the cerebellum with the cerebrum in BD	FC	128 patients with BD; 83 healthy controls	↑ FC motor control areas and emotion; ↓ FC in region related to language production
BD versus MDD				
Dvorak et al. (2019)^54^	Comparison of brain network properties of euthymic MDD, euthymic BD and HC	rsFC	20 patients with BD; 15 patients with MDD; 30 healthy controls	Distinctions in frontal, temporal, and subcortical nodes in emotion control regions such as the limbic system in BD compared with MDD and HCs
Goya‐Maldonado et al. (2016)^55^	To characterize specific alterations in networks such as frontoparietal, cingulo‐opercular, and default mode in BD and MDD	rsFC	20 patients with BD; 20 patients with MDD; 20 healthy controls	↑ rsFC in the frontoparietal network, a central executive and externally‐oriented network in BD
Rive et al. (2016)^56^	Assessment of the distinct structural and functional alterations on medication‐free MDD and BD patients	rsFC	49 patients with BD; 42 patients with MDD	Gray matter volumes of emotion regulation regions and the rsFC of the DMN may be used to categorize MDD and BD (69.1% prediction accuracy)
He et al. (2018)^57^	Assessment of functional connectivity of the cerebellum with the cerebrum in BD and MDD	rsFC	32 patients with BD; 33 patients with MDD; 43 healthy controls	↓ Positive rsFC between the left precuneus and the left cerebellar lobule IX and ↓ negative rsFC between the right subgenual anterior cingulate cortex and the cerebellar vermis in BD and MDD compared with the HC
Chen et al. (2018)^58^	Assessment of the distinct structural and functional alterations in MDD and BD patients	VBM, FC	43 patients with BD; 36 patients with MDD; 79 healthy controls	↓ rsFC between the ACC_L and the left orbitofrontal cortex (OFC_L) in BD and MDD. ↓ rsFC between the SFG_L and the HIP_L in MDD group
Fateh et al. (2019)^59^	Investigation of the hippocampal rsFC analyses to distinguish MDD from BD	rsFC	30 patients with BD; 29 patients with MDD; 30 healthy controls	↑ rsFC of the bilateral anterior/posterior hippocampal regions with lingual gyrus and inferior frontal gyrus (IFG) in BD. ↓ rsFC between the right posterior hippocampus and right IFG, ↑ rsFC between the right anterior hippocampus and lingual gyrus in BD and MDD
Han et al. (2020)^60^	Investigation of specific abnormalities in network flexibility during the configuration of dynamic networks in individuals with MDD and BD	rsFC	40 patients with BD; 61 patients with MDD; 61 healthy controls	↓ rsFC of the left precuneus, bilateral parahippocampal gyrus, and bilateral dorsal medial prefrontal cortex in BD and MDD patients
Luo et al. (2021)^61^	Examine common and unique patterns of aberrant dynamic brain integration and segregation in individuals with MDD and BD	rsFC, ALFF	106 patients with BD; 114 patients with MDD; 130 healthy controls	↓ rsFC between the bilateral PCC/precuneus and the left inferior parietal lobule was reduced in BD and MDD
Chen et al. (2022)^62^	Identifying brain similarities and differences between BD and MDD using whole‐brain regional cerebral blood flow (CBF) and intrinsic FC	CBF, FC	88 patients with BD; 95 patients with MDD; 96 healthy controls	↓ FC between the left inferior frontal gyrus (IFG) and the left posterior lobe of the cerebellum in BD
Goldman et al. (2022)^63^	To study differences in FC between patients with BD2 and MDD using the graph theory approach	ICD	28 patients with BD; 20 patients with MDD; 111 healthy controls	↑ ICD left‐sided frontal, insular, and medial temporal in BD2 and MDD. Frontal, basal ganglia, and fusiform regions showed ↑ right‐sided ICD increases in BD2. ↑ FC between frontal regions of the two hemispheres in BD
BD versus SCZ				
Jimenez et al. (2019)^64^	Examination of functional connectivity within nine RSNs across the cortex via nonparametric permutation testing	rsFC	46 patients with BD; 48 patients with SCZ; 48 healthy controls	Impaired visual network connectivity in BD compared with SCZ
Karcher et al. (2019)^65^	To clarify whether corticostriatal connection pattern crosses diagnostic boundaries and manifests as psychotic bipolar illness	FC	40 patients with BD; 77 patients with SCZ; 60 healthy controls	↓ FC between the putamen, the medial prefrontal cortex and salience network transdiagnostic
Chen et al. (2024)^66^	Identification of neural biomarkers for BD and SCZ	rsFC, sMRI, and DTI	31 patients with BD; 39 patients with SCZ; 43 healthy controls	↑ thickness of the left cuneus cortex in SCZ and BD; ↓ rsFC in the left supramarginal gyrus; ↓ FA in the left superior fronto‐occipital fasciculus

Abbreviations: ALFF, amplitude of low‐frequency fluctuation; BD, bipolar disorder; FC, functional connectivity; MDD, major depressive disorder; rsFC, resting‐state functional connectivity.

Another study on selective resting‐state networks related to executive functions and social cognition, such as the DMN—particularly the connection between the MPFC and posterior cingulate cortex (PCC)—and the sensory‐motor network, showed deficits in BD patients' FC when compared with HC. Furthermore, it became clear that changes in prefrontal connections played a major part in explaining the cognitive deficiencies seen in BD patients.^67^

Zhao et al. investigated the interhemispheric FC (during resting state) in patients with remitted BD. In the middle frontal and precentral gyrus, BD patients exhibited poorer voxel‐mirrored homotopic connectivity (VMHC) compared with HC patients. The results indicated a significant deficit in interhemispheric coordination in individuals with BD.^43^ According to one study BD patients in mania had substantially higher FC between parietal, occipital, and frontal nodes within the dorsal attention network (DAN) than did euthymic patients or HC subjects. Connectivity between dorsal frontal nodes and the rest of the DMN distinguished between mood states and diagnosis.^68^

Some convergent findings should be highlighted: a number of recent studies point to the vermis as a potential biomarker for the prediction of BD. According to recent findings, the cerebellum may have a compensatory function in BD, and when it is unable to perform this function, mania and depression may occur. In their study, Saleem et al., found differences in FC between the fields responsible for control of emotion, motor function, and language and the cerebellar vermis. This connection was stronger in areas related to motor control and emotion and weaker in regions related to language production.^53^

Another research supports the hypothesis that there are changes in cerebello–cerebral connection in BDI and BDII. Patterns of altered cerebello‐cerebral FC were observed in both BD subgroups as compared with HC. Anterior limbic network areas, especially linked to BD's hypomanic states, were affected by impaired dentate–cerebral connection. The concept that cerebello‐cerebral FC variations reflect the neural correlate of subthreshold symptoms, as trait‐based pathology and/or compensatory mechanism to maintain a state of euthymia, is supported by the fact that these altered FC patterns remain during euthymia.^48^

Furthermore, a study by Li et al., examined the rsFC of the anterior and posterior vermis. The study found that both the rsFC between the anterior vermis and the middle cingulate cortex, and the rsFC between the entire vermis and the ventral prefrontal cortex (VPFC), were significantly lower in the BD group compared with the HC group.^46^ Another study demonstrated atypical cerebellum–cerebrum FC in BD patients in comparison with MDD and HCs. Both BD and MDD patients showed weaker positive connectivity between the left precuneus and the left cerebellar lobule IX and weaker negative connectivity between the right subgenual ACC and the cerebellar vermis in comparison to the HC. Moreover, compared with both MDD and HC, BD patients exhibited reduced positive connectivity in the right dorsolateral prefrontal cortex‐left cerebellar lobule Crus I circuit.^57^

Amplitude of low‐frequency fluctuations (ALFF) is a feasible method to look at chronic brain dysfunction in BD.^47^, ^69^, ^70^ Findings highlight the critical role of the right striatum in BD patients' baseline brain function and suggest that aberrant spontaneous brain activity in the cortical–striatal neural circuits is a trait‐like variation in BD.^41^, ^47^ The prefrontal–limbic networks and associated striatal systems are dysfunctional in BD.^69^ Among BD sufferers, ALFF in the left posterior insula (l‐PI) and left superior parietal lobule was significantly decreased. In addition, compared with the MDD group, they had higher ALFF in the right dorsal anterior insula (r‐dAI). These findings provide further evidence that insular subregions could play a role in the fine distinction between BD and MDD.^70^

There is growing body of evidence that differences in FC between individuals with MDD and BD could also be used to distinguish these diagnostic entities.^58^, ^60^, ^61^, ^62^ A recent publication comparing FC in patients diagnosed with these two conditions provided the following results: compared with MDD patients, BD patients show higher FC of the bilateral anterior/posterior hippocampal regions with the lingual gyrus and inferior frontal gyrus (IFG). On the other hand, patients with BD and MDD exhibited a decreased FC between the right posterior hippocampus and right IFG along with enhanced FC between the right anterior hippocampus and lingual gyrus in contrast to HCs.^59^ Another study observed notable changes in network integration between BD and MDD patients, as well as HC, specifically between frontal, temporal, and subcortical nodes in emotion regulation areas such the limbic system and related regions.^54^

The FC differences between MDD and BD were also proven in a large‐scale brain networks study. Increased FC in the FPN was the driving force for differences in bipolar patients. On the other hand, unipolar patients showed decreased connectivity from the cingulo–opercular network to default mode areas and increased FC in the DMN.^55^ According to multimodal study, the gray matter volumes of emotion regulation regions and DMN FC might be used to classify individuals with MDD and BD with 69.1% prediction accuracy.^56^ Another report by Goldman et al. on graph theory analyses aimed to differentiate between MDD and BD in young adults. They discovered that interhemispheric and right‐sided dysconnectivity may serve as distinguishing factors for bipolar depression.^63^

Research exploring transdiagnostic criteria for BD and SCZ revealed corticostriatal dysconnectivity, including decreased association between the putamen and the MPFC, as well as diminished SN connectivity.^65^ As indicated in various publications, hypoconnectivity in the SN is of significant importance in psychotic conditions. Another study identified several key biomarkers for both BD and SCZ, including lower regional functional connectivity strength (rFCS) in the left supramarginal gyrus and increased thickness of the left cuneus cortex.

Furthermore, it has been proposed that BD is specifically indicated by decreased FA in the left superior fronto‐occipital fasciculus, whereas SCZ may be specifically characterized by decreased rFCS in the left inferior parietal region. This approach served as means to differentiate between the two disorders through a multiclass classification model based on multimodal neuroimaging data reaching accuracy of about 70%.^66^ Differences were observed in the sensorimotor network,^50^ medial, and lateral visual networks among BD and MDD patients.^64^ When compared with controls, patients with BD demonstrated noticeably reduced FC homogeneity values in the left middle temporal gyrus (MTG). There were fewer functional connections between the left MTG and the following clusters in BD patients: cluster 1 (left superior temporal gyrus, extend to MTG, rolandic operculum) and cluster 2 (right postcentral, extend to right precentral).^51^

Moreover, in task‐related fMRI, BD patients had higher levels of FC between the MPFC and PCC during positive rumination, as opposed to distraction, and during rest compared with HC. Additionally, after engaging in positive rumination, they displayed increased activity in the MPFC and PCC during processing of positive traits. Patients with BD demonstrated higher levels of FC between the PCC and inferior parietal lobule than HC both at rest and during negative rumination as opposed to distraction.^52^

In another task‐related fMRI study, BD patients exhibited a lack of activation differences in the amygdala and showed reduced FC between the amygdala and ventromedial prefrontal cortex (vmPFC) compared with HCs, regardless of awareness level and type of emotion. When presented with angry and neutral faces compared with blank ovals, BD patients demonstrated greater activation in the medial frontal gyrus. These findings suggested that, independent of awareness level, abnormal amygdala‐vmPFC connection, and brain dysfunction in regions related to emotion appraisal and expression (such as the middle frontal gyrus), may serve as the pathophysiological correlates of emotional processing in BD.^42^

The amygdala connectivity in BD is widely investigated proven to be abnormal by several studies with convergent findings.^44^, ^71^, ^72^, ^73^, ^74^ There is evidence that the SN (amygdala) FC could be used to assess the current suicide risk in BD patients, as a negative correlation existed between current suicide risk and a decrease in FC of the SN.^45^

### 4.3 Effective connectivity

Only seven articles on effective connectivity from the last 10 years were included in this review. Table 3 provides a detailed description of the studies, most of which used methods such as granger causality and dynamic causal modeling (DCM) with samples of 100–200 participants with BD and healthy controls. In an earlier study, a pattern of faulty or decreased connection between the dorso‐lateral prefrontal cortex (DLPFC) and amygdala, which may reflect bipolar individuals' aberrant mood and emotion regulation was reported.^75^ Later research confirmed these findings, showing a difference in amygdala connectivity in BD patients. The effective connectivity from the DLPFC to the amygdala exhibited a difference in the modulatory effect of reappraisal between BD patients and HCs, with BD patients displaying a weaker modulatory influence on this connection compared with HCs. According to the authors, inadequate prefrontal control is indicated by the disruption in BD patients' effective connection from the DLPFC to the amygdala during reappraisal.^78^

**TABLE 3:** Effective connectivity.

Author, year	Aim	Method	Population	Main results
BD versus HC				
Radaelli et al. (2015)^75^	Investigation of the effective connectivity in a sample of patients with BD	DCM	52 patients with BD; 40 healthy controls	DLPFC and ACC—strong reciprocal connections with the amygdale; abnormal or ↓ EC between DLPFC and amygdala
Breakspear et al. (2015)^76^	Hypothesis that functional impairment of the inferior frontal gyrus in those at genetic risk of BD reflects the dysfunction of broader network dynamics underlying the coordination of emotion perception and cognitive control	DCM	55 patients with BD; 41 first‐degree relatives of patients with BD; 45 healthy controls	Relationships between emotion and cognitive control in those at high genetic risk for BD
Roberts et al. (2016)^77^	Study of structural connectivity differences between these groups, with a focus on highly connected hubs and networks involving emotional centers	DWI	38 patients with BD; 84 unaffected HR individuals with at least one first‐degree relative with BD; 96 healthy controls	↓ EC in a right‐sided subnetwork that involved connections between temporal and fronto‐temporal areas in BD; weaker structural brain networks involving key emotional centers in BD
Zhang et al. (2018)^78^	Investigation whether BD patients show abnormal effective connectivity from the prefrontal areas to the amygdala during effortful ER (reappraisal)	DCM	23 patients with BD; 17 healthy controls	↓ Negative affect ratings following reappraisal compared with attending negative pictures; ↓ modulatory effect of reappraisal on the connectivity from the DLPFC to amygdala in BD
Zhang et al. (2022)^79^	Investigation of the effective connectivity difference of the three brain networks (executive control network [ECN], salience network [SN], and default mode network [DMN]) between all patients with BD and healthy controls and between euthymic BD and depressed BD	spDCM	65 patients with BD; 85 healthy controls	Altered EC within the ECN and SN, and between these two networks and the DMN. ↓ inhibitory effects from the SN to the ECN and DMN and ↑ excitatory effects within the ECN, in euthymic BD in contrast to those with depressed BD
BD versus MDD				
Rai et al. (2021)^80^	Investigation of fronto‐parietal (FPN), SN, and DMN networks	ICA	38 patients with BD; 39 patients with MDD; 39 healthy controls	↑ EC between the subgenual cingulate cortex (DMN) and right dorsolateral prefrontal cortex (FPN) in BD relative to MDD and HCs; ↑ EC between the DMN and inferior frontal gyrus, an FPN region in BD relative to MDD
Between groups comparisons				
Kandilarova et al. (2021)^81^	Examine quantitative or qualitative differences in the connectome between psychiatric patients and healthy controls and to delineate the connectome features of MDD, SCZ, and BD	spDCM	101 subjects, divided into four groups: healthy controls and patients with SCZ, BD, or MDD	Mood disorders and SCZ differ in ways depending on certain connectome traits

Abbreviations: BD, bipolar disorder; DCM, dynamic causal modeling; DLPFC; dorso‐lateral prefrontal cortex.

Emotional processing, executive functioning, and cognitive control circuits were disproportionately affected by structural, functional, and effective connectivity disruptions linked to BD. Additionally, compared with both matched control groups and those with BD, people at high risk for BD have different patterns of connectivity. These differences may indicate neurobiological markers of both resilience and risk.^16^

The at‐risk cohort of another study exhibited a distinct variation in the anterior cingulate's hierarchical influence on the effective connectivity between the dorsolateral prefrontal cortex and the IFG. This network disturbance was compensated for by nonspecific, nonhierarchical methods. In people with a high hereditary risk of BD, a unique network disturbance was identified suggesting malfunction in the processes supporting hierarchical interactions between emotion and cognitive regulation.^76^

In another study, compared with matched controls, participants with a high risk of BD had enhanced connectivity in a right lateralized limbic subnetwork and decreased structural connection in two lateralized subnetworks centered on bilateral inferior frontal gyri and left insular cortex. BD was characterized by hypoconnectivity in a small right‐sided subnetwork that connects fronto‐temporal and temporal areas.^77^ These findings can be used not only in a diagnostic process of BD but also to establish the risk for future development of the disorder in relatives of patients with BD and to distinguish the disorder from other mental diseases.

Convergent findings indicate the following trends: when comparing individuals with BD to healthy controls, fMRI reveals aberrant activation in three key brain networks—the SN, executive control network (ECN), and DMN. A recent study based on resting‐state fMRI demonstrated abnormal activation in the three following major neural networks: SN, ECN, and DMN in patients with BD compared with healthy controls. Individuals with euthymic BD had reduced inhibitory effects from the SN to the ECN and DMN and greater excitatory effects within the ECN, in contrast to individuals with depressed BD.^79^

Similar results were found in a study, conducted on MDD and BD patients and healthy controls, showing differences in the ECN and SN not only between HCs and patients with BD but also between BD and MDD. In comparison to MDD and controls, seed‐based studies revealed a considerably higher level of connectivity between the right dorsolateral prefrontal cortex (PFC) and subgenual cingulate cortex (DMN) in BD. These findings imply that connectivity between these networks may be able to differentiate between the two illnesses and may represent a characteristic mechanism in BD that may exist even in the absence of symptoms.^80^

Moreover, SCZ patients were distinguished from those with BD and psychosis by exhibiting greater fluctuation in the effective connectivity between the SN and ECN, as well as a higher clinical severity of disorganization.^82^ Severe mental diseases may be caused by dysfunctions in the SN's self‐regulation, but mood disorders and schizophrenia differ in other important connectome aspects that may serve as possible imaging biomarkers as found in a study using machine learning.^81^

## 5 DISCUSSION

Several trends of abnormal structural, functional, and effective connectivity were identified in studies of BD patients and HCs published over the past 10 years. Trends in structural connectivity showed differences between patients and healthy controls in frontal gyrus, both in ACC and PCC, as well as in emotion and reward networks. FC between cerebellar vermis and the cerebrum was consistently found to be abnormal in BD patients in more than five independent studies. Abnormal FC from several brain regions to the amygdala suggests differences in SN activation in BD compared with healthy controls, a hypothesis supported by effective connectivity findings as well. In addition, effective connectivity studies showed alterations in DMN, SN, and ECN, which can also be linked to the clinical symptoms of mood disorders.

There are several challenging caveats to be outlined on methodological level in connectivity research. One major concern is the validity of the diagnostic procedures in psychiatry. Most psychiatric diagnoses are determined in top‐down fashion, that is from clinical assessment to neuroimaging investigations, where the latter are considered as auxiliary markers. The clinical diagnosis is exclusively based on clinical interviews, which are comprised by subjective evaluations. This raises a large‐scale conceptual debate about psychiatric validity in general.^83^ One possible alternative approach to overcome this caveat is the application of artificial intelligence and machine learning procedures, which can capture both clinical assessments, and connectivity measures.^84^, ^85^

Alternatively, there is the possibility to apply bottom‐up, unsupervised machine learning solely based on neuroimaging data, which represents a highly controversial approach, because it ignores the psychopathological evaluation, which is the essential reason for referral of the patients. The other caveats are the relatively small sample size in most included studies and divergent analytical methods with the later undermining replicability of the results. This calls for standardization of the data processing methods and further integration of clinical and neuroimaging resources by artificial intelligence.

In other words, that would mean semiunsupervised approach for machine learning, where the current diagnostic measures are taken into consideration together with the connectome and other biological data.^86^ This approach has seen increasing utilization in fMRI studies in the recent years. The method enables dissociation from diagnostic classification in psychiatry by including clinical data and connectivity as variables with blinded diagnoses while biological markers remain.

Gabriele Lohmann et al. used a similar approach in their work while predicting neuromarkers for cognitive abilities using fMRI. “Semi‐blind machine learning (SML)” operates under the presumption that individuals in both the training and test sets have access to additional information, such as their educational background. A key aspect of this method is its inclusion of a component specifically designed for bias control.^87^ Cross‐Validation of Functional MRI and Paranoid‐Depressive Scale study uses a two‐step hierarchical approach employing a semisupervised method. Unlike earlier methods based on diagnostic criteria, this enables the discovery of underlying biological processes and the identification of those predictive of diagnostic groupings.^88^ Traditional neuroimaging techniques depend on group averages based on homogeneous patient groups. These methods may enhance variability and cause differences in the literature if subgroups are present, potentially masking variations across different groupings. To address this issue, unsupervised machine learning was utilized in a task‐based fMRI study on OCD patients to identify patient subgroup clusters.^89^

Summarizing the findings of the current review, we can conclude the following: the great majority of structural connectivity research produced divergent results. BD patients showed a reduced left occipital and right parietal clustering coefficient in comparison to healthy individuals. The discovery of anomalies in the reward‐related and emotion‐regulatory subsystems in BD patients represents a converging trend. Compared with HCs, BD patients have longer typical path lengths, a decrease in the left frontotemporal network connections, as well as greater rich‐club dysconnectivity.

The majority of studies on FC presented divergent results. The temporal–parietal junction's rsFC with the parahippocampus, cerebellum, and fusiform gyrus showed poorer associations with cognitive empathy in BD patients than in healthy controls. Another study found that BD patients had deficits in their FC when compared with HC in specific resting‐state networks related to executive functions and social cognition, such as the DMN (specifically, the connection between the MPFC and PCC and the sensory‐motor network). Some convergent results in FC imply the cerebellum's role in BD. The vermis may be a potential biomarker for BD prediction, according to several studies. Recent research suggests that the cerebellum may play a compensatory role in BD, and that mania and depression may result from its incapacity to carry out this role.

Numerous studies yield divergent findings in terms of effective connectivity, making it impossible to draw a firm conclusion. However, research indicates disruption in BD's emotion‐related networks, particularly those linked to the amygdala. The amygdala is closely connected to both the ACC and the DLPFC, which are key components of emotion regulation.

Disruptions of structural, functional, and effective connections associated with BD have been found to disproportionately impact the circuits responsible for executive functioning, cognitive control, and emotional processing. Convergent findings indicate the following trends: fMRI demonstrated abnormal activation in three major brain networks, the SN, ECN, and DMN, when comparing people with bipolar disease to healthy controls.

To conclude, further research conducted on larger samples is needed to establish the abnormalities in SC, FC, and EC as potential biomarkers and to validate fMRI as a diagnostic tool for BD in the future. Given that many studies achieve an accuracy of around 70%, this digital approach holds promise for providing objective diagnoses, potentially minimizing the subjectivity inherent in current diagnostic methods. A fast and accurate diagnosis through neuroimaging could mitigate the risk of misdiagnosing depressed patients. The use of methods such as semiunsupervised machine learning, may lead to a more accurate diagnostic process based on clinical biomarkers rather than diagnostic classification. Furthermore, identifying rich‐club hubs with numerous abnormal connections to various regions could serve as a crucial point in refining the treatment options through defining the pathophysiology behind the diagnosis and employing targeted neurotransmitters for more effective treatment.

## AUTHOR CONTRIBUTIONS

*Conceptualization*: D.S. *Methodology*: S.K. *Investigation*: T.G. and B.V. *Resources*: D.S. *Writing*—*original draft preparation*: T.G. and B.V. *Writing*—*review and editing*: S.K. and D.S. *Visualization*: T.G. *Supervision*: D.S. *Project administration*: D.S. *Funding acquisition*: D.S. All authors have read and agreed to the published version of the manuscript.

## CONFLICT OF INTEREST STATEMENT

The authors declare no conflicts of interest.

### PEER REVIEW

The peer review history for this article is available at https://www.webofscience.com/api/gateway/wos/peer‐review/10.1111/acps.13742.

## Data Availability

The data that support the findings of this study are available from the corresponding author upon reasonable request.
